# Supplementary material for: Near-field acoustic imaging with a caged bubble
Source: Nat Commun. 2024 Nov 27;15:10275. doi: 10.1038/s41467-024-54693-1 (PMC11603172; doi:10.1038/s41467-024-54693-1)
Supplement: Supplementary file 1 — Supplementary Information [file 41467_2024_54693_MOESM1_ESM.pdf]

Near-field acoustic imaging with a caged bubble
Supplementary Information

Dorian Bouchet[1], Olivier Stephan[1], Benjamin Dollet[1], Philippe Marmottant[1], and Emmanuel Bossy[1]

[1] *Université Grenoble Alpes, CNRS, LIPhy, 38000 Grenoble, France*

S1 – Temporal drift of the resonance frequency

During an acquisition, the volume of air in a bubble tends to slightly decrease over time, leading to a slight increase of the resonance frequency of the bubble over time. To characterize this effect, we present in Fig. S1 the resonance frequency of different bubbles in the absence of sample, as a function of time. All measurements were performed with the same 3D-printed cage, but due to a variability in the process of bubble formation by immersion of the cage, each bubble is characterized by a slightly different volume, resulting in different resonance frequencies. Moreover, air is progressively lost by the bubbles over time, resulting in a slow increase in the observed resonance frequencies. Note that the rate at which air escapes the cage can also vary, which may arise due to variations in the wettability of the cage, that is not precisely controlled during the immersion of the cage.

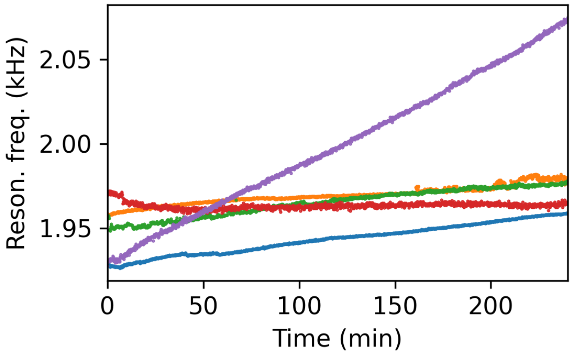

Supplementary Figure S1 — Temporal drift of the resonance frequency. Resonance frequency of different bubbles in the absence of sample, as a function of time. The different colors represent different measurements. Before each measurement, the 3D-printed cage is dried before being immersed into water. A new bubble is thus formed inside the cage prior to each measurement. For each measurement, there are small variations in the amount of air trapped into the cage, resulting in slightly different resonance frequencies.

This effect can be significant on long acquisitions, such as those presented in Fig. 4 of the manuscript. For the image of the Eiffel tower (acquisition time of 1 hour and 58 min), the reso-

nance frequency of the bubble in the vicinity of steel increased from 1.78 kHz to 1.79 kHz over the whole acquisition. For the image of the SNAM acronym (acquisition time of 1 hour and 49 min), the resonance frequency of the bubble in the vicinity of steel increased from 1.69 kHz to 1.79 kHz over the whole acquisition. To correct for this effect, we simply assume that the resonance frequency varies linearly in time, and we apply a correction to all measured resonance frequencies, taking as a reference the central frequency extracted from the power spectral density measured at the beginning of the experiment (i.e., the first point of the scan). The uncorrected images are presented in Fig. S2. While this correction does not significantly affect the reconstructed image of the Eiffel tower, we can see that it does affect the image of the SNAM acronym. On the uncorrected image of the SNAM acronym sample (Fig. S2c), the resonance frequency is indeed higher for the data points that have been measured at the end of the acquisition (lower right corner) as compared to those that have been measured at the beginning of the acquisition (upper left corner). Overall, this strategy allows performing long acquisitions with the same bubble (up to dozens of hours).

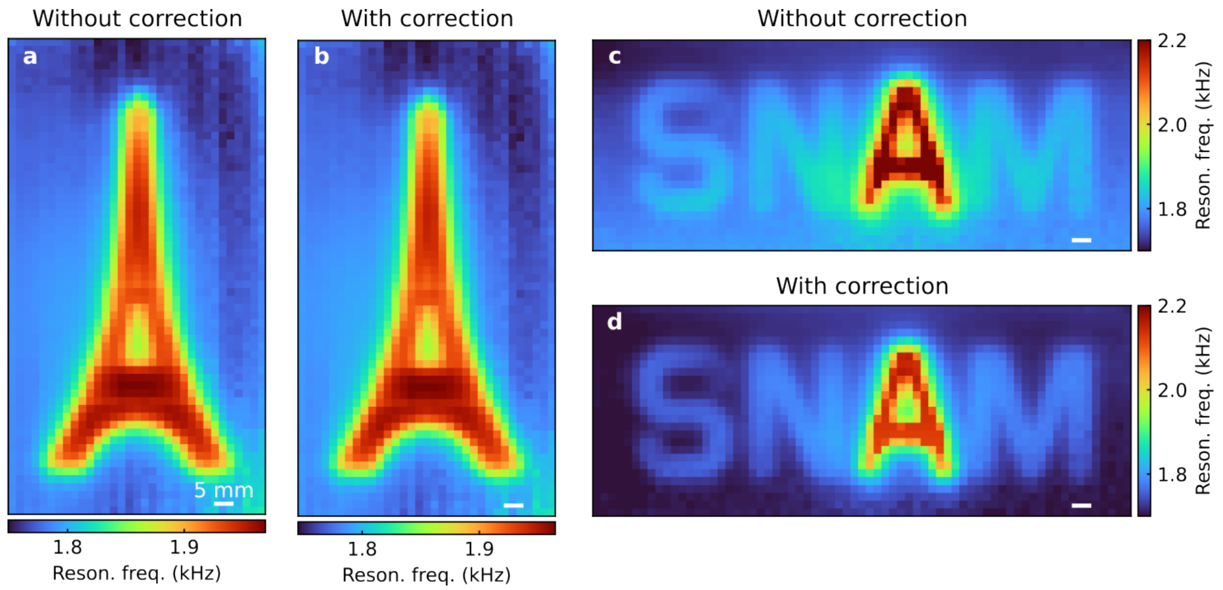

Supplementary Figure S2 — Correction applied on the frequency images. Images of the measured central frequencies, before (**a**,**c**) and after (**b**,**d**) correcting for the slow decrease in the gas volume inside the bubble. The correction simply consists in measuring the frequency shift during the acquisition from measurements of the same data point before and after the acquisition, and performing a linear interpolation to estimate the frequency shift during the acquisition. Scale bar: 5 mm.

S2 – Finite-difference time-domain simulations

S2.1 – Bubble dynamics close to interfaces for spherical and cubic bubbles

FDTD results that we presented in the manuscript were normalized: the resonance frequency was normalized by the homogeneous-space resonance frequency, and the bubble-to-interface distance was normalized by the effective bubble diameter. In this section, we present non-normalized results,

in order to compare the resonance frequency of spherical and cubic bubbles of identical volume. Simulations with cubic bubbles were performed with a length size $a_0 = 2.5\,\text{mm}$ ($16 \times 16 \times 16 = 4096$ pixels, mesh size = 0.15625 mm, volume of $15.625\,\text{mm}^3$). Simulations with spherical bubbles were performed with a the same exact volume. Note that because of the discretization over a Cartesian mesh, matching the volume of an approximate discretized sphere to an exact value required us to slightly tune the mesh size: our discretized sphere had 4032 pixels, imposing a mesh size of 0.15707 mm in order to reach a volume of $15.625\,\text{mm}^3$.

Results are presented in Fig. S3, for both a spherical bubble surrounded by perfectly matched layers and a cubic bubbles inside a finite tank. The resonance frequency observed for the spherical bubble surrounded by perfectly matched layers almost exactly matches the one obtained using the expression derived by Morioka based on the potential flow of an incompressible liquid[1], which describes the case of a spherical bubble close to an interface separating two semi-infinite media. This evidences that neglecting the compressibility of the flow is an excellent approximation in the near-field of the bubble, as our FDTD simulations do take into account the finite compressibility in the fluid. In comparison, the resonance frequency observed for the cubic bubble in the finite tank is slightly larger (approximately 2.3%). A similar effect has been observed in a previous work[2], in which a 2.6% increase of the homogeneous-space resonance frequency was reported for a cubic bubble as compared to a spherical one. This indicates that the different bubble geometry is the dominant effect to explain the small increase in the resonance frequency observed for cubic bubbles. The influence of the finite tank is smaller in comparison, as we further verified by conducting additional numerical simulations with cubic bubbles surrounded by perfectly-matched layers (no significant differences were observed as compared to the case of the cubic bubble in the finite tank).

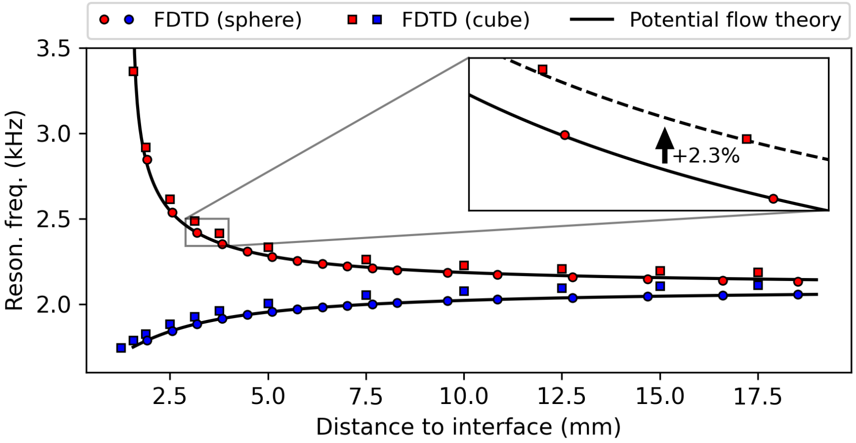

Supplementary Figure S3 — Resonance frequency for spherical and cubic bubbles. Relation between the resonance frequency and the bubble-interface distance for rigid (in blue) and free (in red) interfaces. Theoretical predictions obtained using Eq. (2) of the manuscript are represented by black lines. Results of FDTD simulations performed with a spherical bubble in water surrounding by perfectly-matched layers are represented by blue and red circles. Results of FDTD simulations performed with a cubic bubble in water enclosed in a finite tank are represented by blue and red squares. These simulations have been performed so that the volume of the spherical bubble matches the volume of the cubic one.

S2.2 – Instantaneous pressure fields around spherical and cubic bubbles

At any given time in the FDTD simulations, we can record a snapshot of the pressure field around the bubble. In Fig. S4, we show the normalized pressure field around cubic and spherical bubbles, in the vicinity of rigid and free interfaces. These snapshots were recorded at the first maximum of the oscillating field after the exciting field has vanished. Note that other times could have been chosen, as the normalized pressure field is essentially the same at any given time after the exciting field has vanished.

On these snapshots, it clearly appears that the field is affected by the detailed geometry of the bubble only in its very close vicinity, and that the fields generated by cubic and spherical bubbles are very similar as soon as they are evaluated at distances larger than the bubble size. In this regime, we can conclude that the fields are essentially those that would be generated by two in-phase monopoles (rigid interface) or two out-of-phase monopoles (free interface).

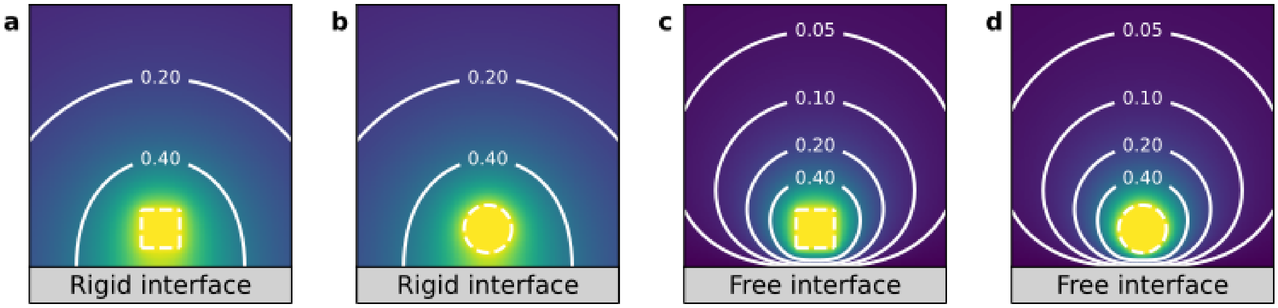

Supplementary Figure S4 — Pressure fields around spherical and cubic bubbles. **a**,**b**, Normalized instantaneous pressure fields calculated by FDTD around a cubic bubble **a**, and a spherical bubble **b**, close to a rigid interface. **c**,**d**, Analogous to **a**,**b** for bubbles close to a free interface. On these figures, the edges of the bubble are represented by dotted white lines, and the edge size of the whole field of view is 17.2 mm.

S3 – Multispectral images

In the manuscript, we described two imaging modes that provide different image contrasts, namely, intensity imaging (see Fig. 4a of the manuscript) and central frequency imaging (see Fig. 4b of the manuscript). However, in the intensity imaging mode, the choice of the frequency at which the image is formed is arbitrary. In the manuscript, we chose to present images measured at a frequency close to the natural resonance frequency of the bubble in water ($f = 1.96$ kHz), but other choices are possible. Thus, for the sake of completeness, we present here the intensity images obtained at various frequencies ranging from 1.48 kHz to 2.40 kHz.

S3.1 – The Eiffel tower sample

In Fig. S5, we show multispectral images measured for the Eiffel tower sample. For frequencies smaller than 1.88 kHz, we observe that the intensity scattered by the bubble is stronger when the bubble is above stainless steel, which is due to the large negative frequency shift experienced by the bubble in these areas. In contrast, for frequencies larger than 1.88 kHz, we observe that the intensity

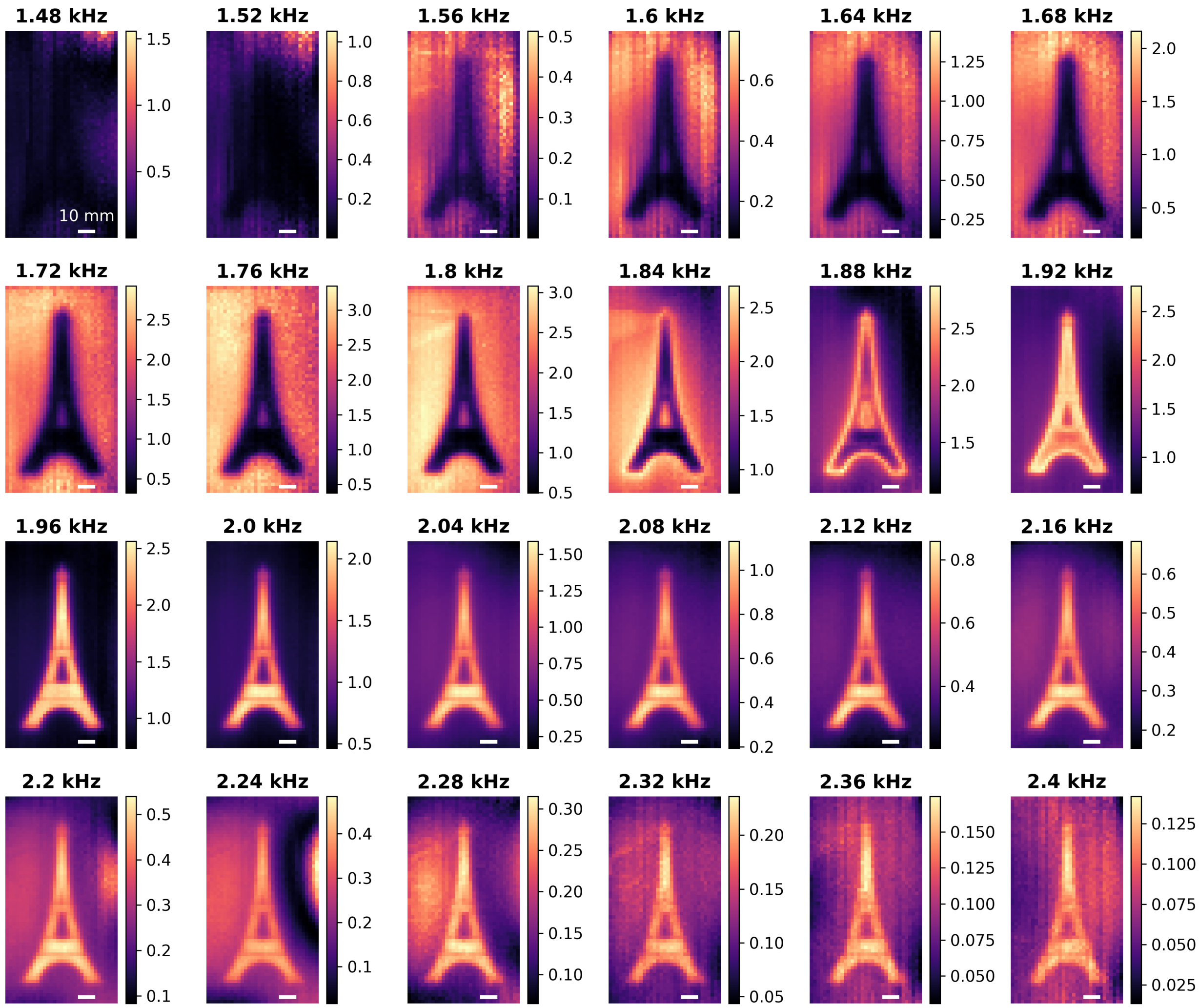

Supplementary Figure S5 — Multispectral intensity images of the Eiffel tower sample. Power spectral density of the scattered field for different frequencies ranging from 1.48 kHz to 2.4 kHz. The contrast of the images is inverted as the imaging frequency is increased: low frequency images highlight the areas above which the resonance frequency of the bubble is lower (i.e., when the bubble is above stainless steel), while high frequency images highlight the areas above which the resonance frequency is higher (i.e., when the bubble is above areas filled with water). Scale bar: 10 mm.

scattered by the bubble is stronger when the bubble is above areas filled with water, for which the resonance frequency is less shifted.

S3.2 – The SNAM acronym sample

In Fig. S6, we show multispectral images measured for the SNAM acronym sample. For frequencies larger than 2.12 kHz, we observe that the intensity scattered by the bubble is stronger when the bubble is above areas filled with air (i.e. the letter "A"), which is due to the large positive frequency shift experienced by the bubble in these areas. For frequencies in-between 1.76 kHz and 2.12 kHz, the intensity scattered by the bubble is stronger when the bubble is above areas filled with water

(i.e. the letters "S", "N" and "M"); in this range, the resonance frequency of the bubble is weakly influenced by its environment. Finally, for frequencies below 1.76 kHz, we observe that the intensity scattered by the bubble is stronger when the bubble is above stainless steel, which is due to the large negative frequency shift experienced by the bubble in these areas.

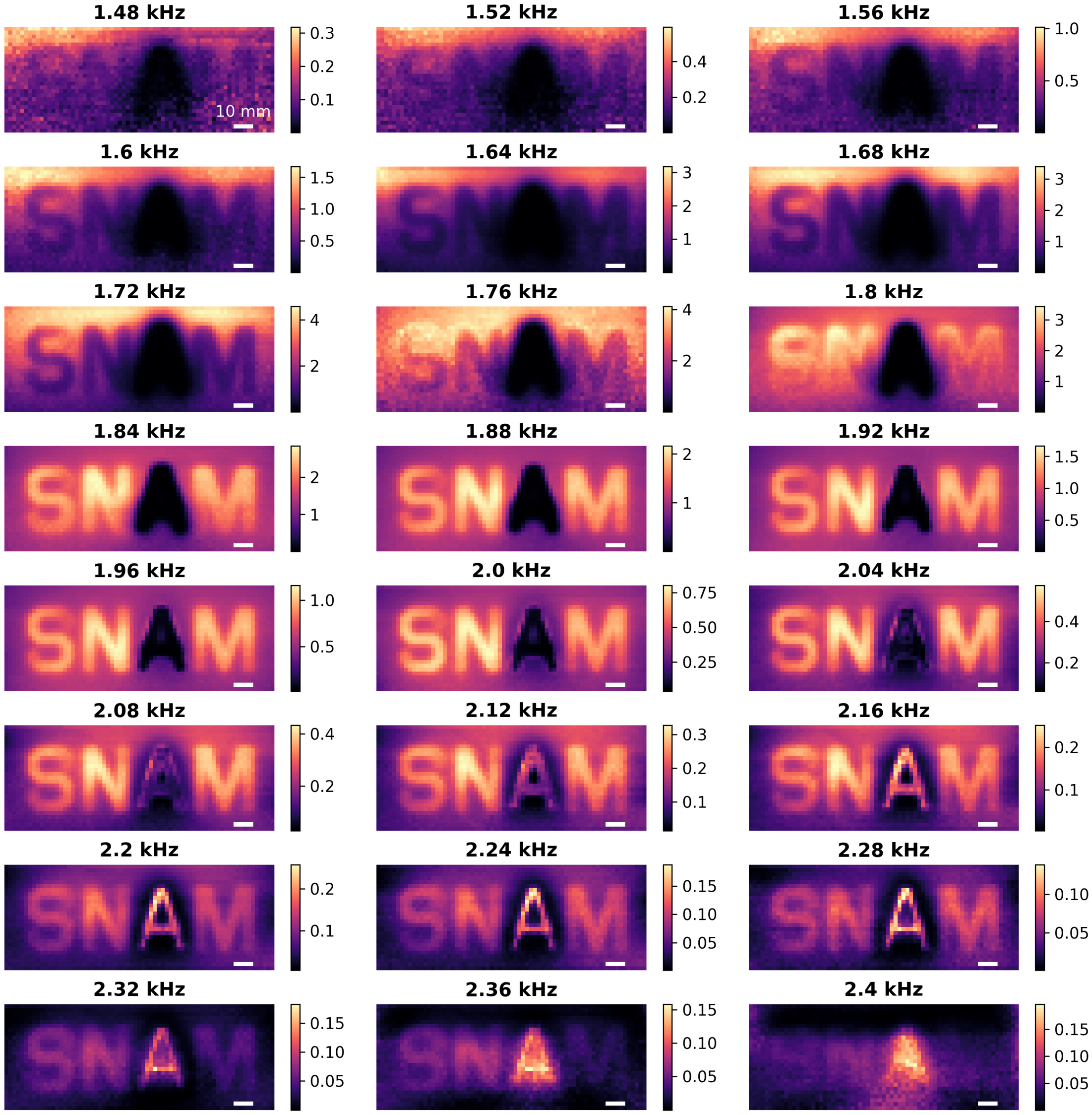

Supplementary Figure S6 — Multispectral images of the SNAM acronym sample. Power spectral density of the scattered field for different frequencies ranging from 1.48 kHz to 2.4 kHz. The letter "A", which is filled with air, is especially visible at high frequencies, as expected for a bubble resonating above a free interface. At lower frequencies, this letter is not visible, as the measured signal scattered by the bubble is very weak above a water-air interface. Scale bar: 10 mm.

S4 – Transverse resolution as a function of the bubble-sample distance

In order to quantify the transverse resolution of the technique, we adopt a strategy inspired by Fourier ring correlation (FRC), which provides a resolution criterion that can be computed directly from experimental data[3–6]. The method is based on n statistically-independent measurements of some raw data taken as a function of spatial coordinates, either in 1D (a line scan) or in 2D (an image scan). Here, we consider as raw data the fields measured in the presence of the bubble during a 1D line scan performed above a resolution test sample composed of three grooves (see Figure 5a of the manuscript). We denote this data set $\phi_{\mathrm{w}}^{(i)}(x,t)$, where i denotes the index of the noise realization. We then successively apply a temporal discrete Fourier transform and a spatial discrete Fourier transform to these data, which results in a data set noted $\hat{\phi}_{\mathrm{w}}^{(i)}(k_x,\omega)$. As an illustration, we show in Fig. S7a two independent measurements extracted from this data set, taken for a bubble-sample distance $z = 2.5\,\mathrm{mm}$. We observe that, while noise weakly affects low spatial frequencies, it strongly corrupts high spatial frequencies.

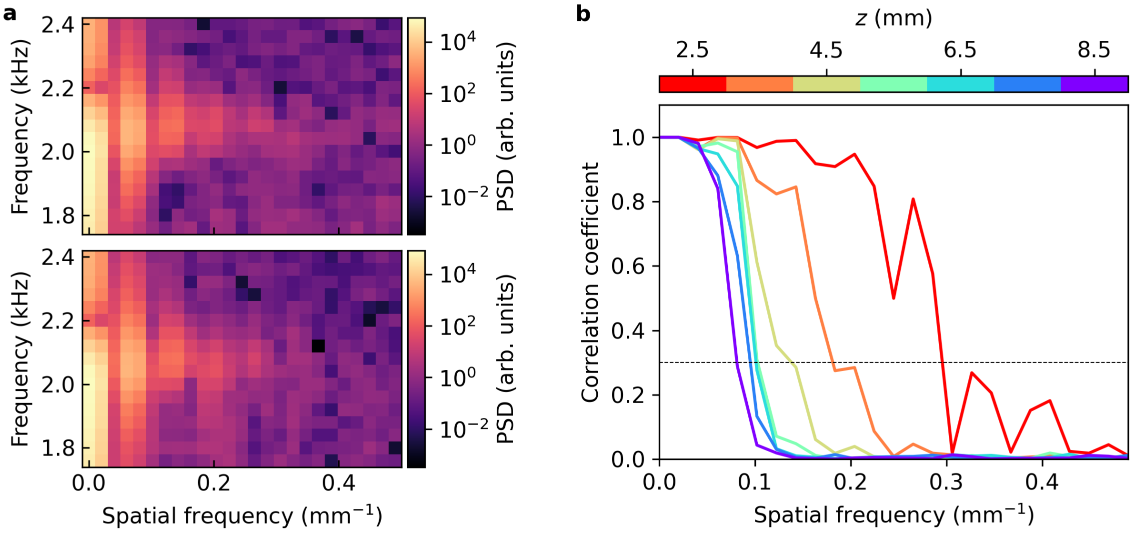

Supplementary Figure S7 — Determination of the transverse resolution of the approach. **a**, Power spectral density measured in the presence of the bubble, with the temporal frequency in the vertical axis and the spatial frequency in the horizontal axis. This was obtained by Fourier transforming the field measured during a 1D line scan performed above a resolution test sample composed of three lines (see Figure 5a of the manuscript). The top and bottom sub-figures represent two identical measurements performed in the presence of noise. Noise weakly affects low spatial frequencies, but strongly corrupts high spatial frequencies. **b**, Absolute value of the mean correlation coefficient calculated by correlating the power spectral densities measured for each spatial frequency. For each distance z, the signals have been measured 200 times with different noise realizations, and the average has been calculated over 19900 different pairs. By setting an arbitrary threshold (here equal to 0.3), we can calculate the maximal spatial frequency ξ contained in these 1D line scans (see Figure 5b of the manuscript).

By calculating the correlation coefficient between such measurements as a function of k_x, we can assess which spatial frequencies are robust to noise fluctuations, and which spatial frequencies are

dominated by noise. We use the following expression for the mean correlation coefficient:

$$C(k_x) = \frac{1}{n(n-1)/2} \sum_{(i,j)\in\mathcal{P}} \frac{\sum_{l\in\mathcal{Q}} \hat{\phi}_{\mathrm{w}}^{(i)}(k_x,\omega_l)[\hat{\phi}_{\mathrm{w}}^{(j)}(k_x,\omega_l)]^*}{\sqrt{\sum_{l\in\mathcal{Q}} |\hat{\phi}_{\mathrm{w}}^{(i)}(k_x,\omega_l)|^2}\sqrt{\sum_{l\in\mathcal{Q}} |\hat{\phi}_{\mathrm{w}}^{(j)}(k_x,\omega_l)|^2}}, \tag{S1}$$

where $\mathcal{P}$ denotes the ensemble of all possible pairs of noise realizations and $\mathcal{Q}$ denotes the ensemble of all temporal frequencies for which the signal coming from the bubble is significant. In practice, the signals have been measured $n = 200$ times with different noise realizations, and thus the average over noise realization has been calculated over $n(n-1)/2 = 19900$ pairs. The sums over temporal frequencies run from 1.75 kHz to 2.45 kHz for these measurements. Finally, note that a Hann window was applied to the data along the spatial dimension before applying the spatial discrete Fourier transform in order to avoid spectral leakage.

The amplitude of the measured correlation coefficient is shown in Fig. S7b for different bubble-sample distances z. We observe that the correlation coefficient is close to unity for small spatial frequencies, and tends to zero for large spatial frequencies. Moreover, larger spatial frequencies are observed for smaller values of z, which supports the observation that the resolution rapidly degrades with the bubble-sample distance. In order to extract a single resolution criterion from these data, we identify the cutoff frequency ξ such that $|C(\xi)| = 0.3$, where the threshold value 0.3 has been chosen arbitrarily to distinguish signal from noise. Note that results only weakly depend of this threshold value, as the correlation coefficient rapidly drops from 1 to 0 for each value of z. Finally, the resolution is simply calculated as the inverse of the observed cutoff frequency ($\mathcal{R} = 1/\xi$).

S5 – Number of measurements per data point

In this section, we compare the signal-to-noise ratio of the measured resonance frequency for different numbers of measurements per data point, taking as an example the 1D line scans performed above the resolution test sample (Fig. 5a of the manuscript). While averaging each data point over 200 measurements yields an excellent signal-to-noise ratio (Fig. S8c), averaging over 20 measurements already gives us a very good signal-to-noise ratio (Fig. S8b). Moreover, it is also possible to rely a single measurement per data point, at the cost of a degraded signal-to-noise ratio (Fig. S8a).

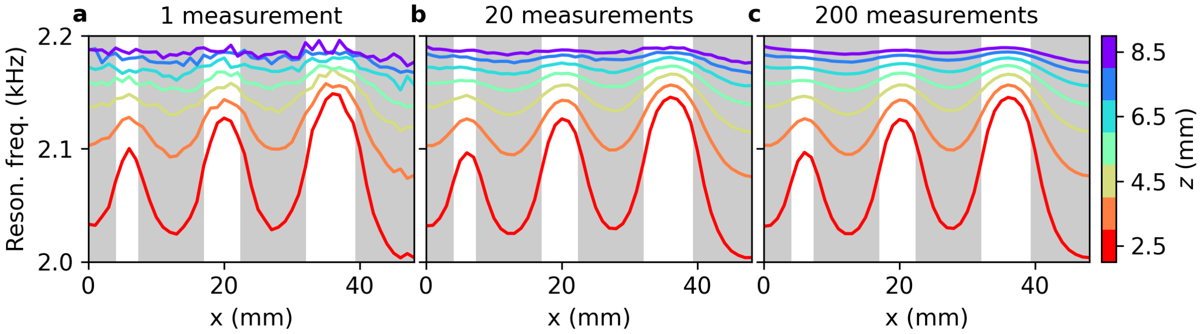

Supplementary Figure S8 — Influence of the number of measurements per data point. Measurements of the resonance frequency of the bubble for the 1D line scans shown in Fig. 5a of the manuscript. The number of measurements used at each point is either 1 (**a**), 20 (**b**) or 200 (**c**).

In practice, in our imaging experiments, each data point was obtained by averaging over 20 mea-

surements; even though this was not strictly necessary, it gave us a better signal-to-noise ratio without strongly affecting the total acquisition time. Indeed, taking the example of the Eiffel tower sample, the total acquisition time was 1 hour and 58 min but, during this time, only around 19 min were spend for actually measuring the data. This clearly shows that the total acquisition time is mostly due to the time spent by the motorized stage to go from position to position, and not by the measurement time.

S6 – Fitting procedure

In Fig. 2d,h of the manuscript, we compare the results of the 1D line scans in the z-direction to the theoretical predictions derived by Morioka[1]. The model function is:

$$f_\pm(\Delta z, d_{\mathrm{eq}}, z_0) = \left(\frac{c_g \sqrt{3\rho_g/\rho_l}}{\pi d_{\mathrm{eq}}} \right) \times \sqrt{\sum_{n=0}^{+\infty} \frac{(\mp 1)^n \sinh(\beta)}{\sinh[(n+1)\beta]}}, \qquad \text{(S2)}$$

where $\cosh(\beta) = 2(z_0 + \Delta z)/d_{\mathrm{eq}}$. In Eq. (S2), the first term (inside braces) corresponds to Minnaert's formula giving the homogeneous resonance frequency, while the second term (under the square root) corresponds to Morioka's result giving the distance dependence. Note that in practice we need to truncate the series for their numerical evaluation; here we keep the first 100 terms, which is sufficient to estimate the series up to machine precision. Taking $c_g = 340\,\mathrm{m/s}$, $\rho_g = 1.2\,\mathrm{kg/m^3}$ and $\rho_l = 1000\,\mathrm{kg/m^3}$, we are left with three parameters: the effective bubble diameter d_{eq}, the bubble-interface distance for the first measurement point z_0 (when the bubble is closest to the interface) and the relative displacement applied by the motorized stage for all other measurement points Δz. While Δz is precisely controlled by the stage in the experiment, it is difficult to precisely control the value of two other parameters d_{eq} and z_0. However, we can estimate these values directly from the data by treating them as free parameters. They are numerically determined by solving

$$d_{\mathrm{eq}}, z_0 = \underset{d', z'}{\operatorname{argmin}} \left(\sum_i \left[f_\pm^{\exp}(\Delta z_i) - f_\pm(\Delta z_i, d', z') \right]^2 \right), \qquad \text{(S3)}$$

where $f_\pm^{\exp}(\Delta z_i)$ denotes the experimental results of the 1D line scans in the z-direction. In the case of the water-steel interface, we obtain an effective diameter of 3.1 mm and a bubble-interface distance z_0 of 2.3 mm. In the case of the water-air interface, we obtain an effective diameter of 3.4 mm and a bubble-interface distance z_0 of 2.6 mm. These values are consistent with what can be expected from the geometry of the cages (volume of trapped air $V \leq 20\,\mathrm{mm^3}$, which gives $d_{\mathrm{eq}} \leq 3.4\,\mathrm{mm}$) and from our procedure to control the minimal bubble-interface distance ($z_0 \simeq 2.5\,\mathrm{mm}$ by establishing the contact by eye and retracting it by 1 mm). The standard error on these values can be roughly estimated from the covariance matrices obtained using `scipy.optimize.curve_fit`; the standard errors for the estimated effective diameters are 0.001 mm (water-steel interface) and 0.0005 mm (water-air interface); the standard errors for the estimated bubble-interface distances are 0.04 mm (water-steel interface) and 0.01 mm (water-air interface).

In practice, varying z_0 amounts to horizontally translate the function $f_\pm$, and varying d_{eq} amounts to vertically translate this function (up to a very good approximation). Therefore, the precise value of these parameters have no effect on the shape of the model function. We can thus conclude that the theoretical model of Morioka, originally introduced for spherical bubbles, accurately describes the shape of the distance dependence that we experimentally observe using square bubbles.

References

1. Morioka, M. Theory of Natural Frequencies of Two Pulsating Bubbles in Infinite Liquid. *Journal of Nuclear Science and Technology* **11**, 554–560 (1974).
2. Boughzala, M., Stephan, O., Bossy, E., Dollet, B. & Marmottant, P. Polyhedral Bubble Vibrations. *Physical Review Letters* **126**, 054502 (2021).
3. Saxton, W. O. & Baumeister, W. The correlation averaging of a regularly arranged bacterial cell envelope protein. *Journal of Microscopy* **127**, 127–138 (1982).
4. Van Heel, M. Similarity measures between images. *Ultramicroscopy* **21**, 95–100 (1987).
5. Nieuwenhuizen, R. P. J. *et al.* Measuring image resolution in optical nanoscopy. *Nature Methods* **10**, 557–562 (2013).
6. Banterle, N., Bui, K. H., Lemke, E. A. & Beck, M. Fourier ring correlation as a resolution criterion for super-resolution microscopy. *Journal of Structural Biology* **183**, 363–367 (2013).
