# Supplementary material for: Near-field acoustic imaging with a caged bubble
Source: Nat Commun. 2024 Nov 27;15:10275. doi: 10.1038/s41467-024-54693-1 (PMC11603172; doi:10.1038/s41467-024-54693-1)
Supplement: Supplementary file 2 — Description of Additional Supplementary Files [file 41467_2024_54693_MOESM2_ESM.pdf]

## Description of Additional Supplementary Files

**Supplementary Movie 1** - Movie illustrating how images are built up when the bubble is scanned over the sample: for each measurement point, the sound heard corresponds to the sound emitted by the bubble. One can note the change in pitch when the bubble is scanned over zones of the sample with different mechanical properties: the sound pitch is higher when the bubble is over water (Eiffel Tower) than when it is over steel.

**Supplementary Movie 2** - Movie illustrating how images are built up when the bubble is scanned over the sample: for each measurement point, the sound heard corresponds to the sound emitted by the bubble. One can note the change in pitch when the bubble is scanned over zones of the sample with different mechanical properties: the sound pitch is the highest when the bubble is over air (letter A), and is the lowest when it is over water (other letters).
